# Supplementary material for: Is There a Difference in Clinical Features, Microbiological Epidemiology and Effective Empiric Antimicrobial Therapy Comparing Healthcare-Associated and Community-Acquired Vertebral Osteomyelitis?
Source: Antibiotics (Basel). 2021 Nov 18;10(11):1410. doi: 10.3390/antibiotics10111410 (PMC8615006; doi:10.3390/antibiotics10111410)
Supplement: Supplementary file 1 [file antibiotics-10-01410-s001.zip › antibiotics-1447534-supplementary.pdf]

Table S1. Predicted efficacy of empiric antimicrobial regimes for the CAVO and the HAVO cohort for mono- and combination therapies. Values are numeric and display the relation between the results for CAVO and HAVO cohorts. Statistically significant differences (*p*-values) are marked in bolt. S: sensitive; R: resistant; X: unknown sensitivity.

	CAVO/HAVO			
	S	R	X	p
Mono-therapy				
Vancomycin	17/18	5/4	1/0	0.489
Meropenem	22/14	1/8	0/0	**0.008**
Gentamycin	16/13	0/5	7/4	0.809
CoAmoxi	21/14	2/7	0/1	**0.026**
Ciprofloxacin	14/14	6/7	3/1	0.691
Clindamycin	14/15	2/5	7/2	0.363
Rifampicin	11/13	0/3	12/6	0.205
PipTaz	22/15	1/7	0/0	**0.017**
Cefazolin	15/12	6/10	2/0	0.653
Cefotaxime	20/14	2/8	1/0	0.095
Ceftriaxone	17/8	2/6	4/8	**0.021**
Linezolid	16/20	5/2	2/0	0.068
Teicoplanin	17/20	5/2	1/0	0.132
Combination therapy				
Meropenem_Vancomycin	23/21	0/1	0/0	0.307
Gentamicin_Vancomycin	22/19	0/3	1/0	0.311
CoAmoxiclav_Vanco	22/19	1/2	0/1	0.269
Vancomycin_Ciprofloxacin	22/19	1/3	0/0	0.279
Gentamycin_Clindamycin	20/16	1/6	2/0	0.328
Vancomycin_PipTaz	23/20	0/2	0/0	0.144
Vancomycin_Cefazolin	17/18	5/4	1/0	0.489
Vancomycin_Cefotaxime	22/20	1/2	0/0	0.528
Vancomycin_Ceftriaxone	21/20	1/2	1/0	1.000
Ciprofloxacin_Rifampicin	19/18	3/3	1/1	0.946
Teicoplanin_PipTaz	23/22	0/0	0/0	1.000
Linezolid_PipTaz	23/22	0/0	0/0	1.000
